# Editorial: Tumor microenvironment immunophenotypes and disease progression

**DOI:** 10.3389/fimmu.2023.1141084

**Published:** 2023-01-24

**Authors:** Ana Carolina Monteiro, Ana Paula Lepique, Martin Bonamino, Mercedes Beatriz Fuertes

**Affiliations:** ^1^ Osteo and tumor immunology laboratory (LOIT), Biology Institute (EGB), Fluminense Federal University (UFF), Rio de Janeiro, Brazil; ^2^ Laboratory on Thymus Research, Oswaldo Cruz Foundation, Rio de Janeiro, Brazil; ^3^ Instituto de Ciências Biomédicas, Universidade de São Paulo, Department of Immunology, São Paulo, Brazil; ^4^ Programa de Carcinogeênese Molecular, National Cancer Institute (INCa), Rio de Janeiro, Brazil; ^5^ Vice-presidência de pesquisa e coleções biológicas, Oswaldo Cruz Foundation, Rio de Janeiro, Brazil; ^6^ Laboratorio de Fisiopatología de la Inmunidad Innata, Instituto de Biología y Medicina Experimental (IBYME), Consejo Nacional de Investigaciones Científicas y Técnicas (CONICET), Buenos Aires, Argentina

**Keywords:** tumor microenvironment (TME), immunophenotypes, tumor-infiltrating immune cells, biomarker, tumor immunity, disease progression

Tumor cells constantly interact with their microenvironment, which comprises a great diversity of immune cells and non-immune stromal cells, such as endothelial cells and fibroblasts. These interactions are considered critical regulators of tumor development, growth, invasion, and establishment of metastases. A variety of immune cells infiltrating the tumor microenvironment (TME) exists across different cancer types but also among patients with the same tumor disease and even in different tumor areas within the same patient. Indeed, their functional immunophenotypes, localization inside or outside the TME, molecular patterns, cytokine signatures, densities, and metabolic status have been implicated in the promotion or inhibition of cancer progression, recurrence, and successful response to immunotherapies. Therefore, studying the immunological phenotypes, “immune contexture”, or immunoscore of the TME is of paramount importance given the clinical impact of their composition and extent. For instance, a strong infiltration by CD8^+^ cytotoxic T cells, Th1 CD4^+^ T cells, type 1 like macrophages (Mϕs) and neutrophils (Nϕs), B cells and follicular helper T cells (TFh) are generally associated with a favorable prognosis with long-term survival and prediction of response to treatment, while the presence of regulatory, Th2 and/or Th17 CD4^+^ T cells, and type 2 like Mϕs and Nϕs infiltrating TME are widely considered negative prognostic markers.

Providing important insights, the past twenty years have witnessed an explosion of research into the biology and clinical applications of tumor immune contexture to restrict and impair tumorigenesis. Accordingly, this Research Topic was developed to update our current knowledge about the complexity and diversity of the immune contexture of TME and its influence in disease progression and response to therapy. Under this Research Topic, a series of articles were published, providing meaningful insights toward this field. Briefly, this collection comprises ten original research articles, and one review of the current literature.

Three of the manuscripts focused on the study of colon cancers for identification of new biomarkers for prognosis. Xu et al. addressed the importance of colon adenocarcinoma classification based on immunophenotypes. These authors described seventeen prognostic-related immune characteristics (including IFNG signature, MDSC and T cell abundance) to cluster colon cancers in three distinct immune signatures (IS) with increasingly better prognostic and therapeutic responses. Briefly, IS1 is immune infiltrated but immunosuppressive, IS2 is immune “cold” and IS3 is immune “hot”. The authors believe that this immune-based classification could be employed as a tool for personalized colon cancer immunotherapy decision and/or tumor prognosis. Wang et al. explored the potential of TME *immunoscore* classification for stage-III colon cancer patients’ prognosis and therapeutics. They identified differentially expressed genes between the high and low TME immunoscore groups and characterized EPSTI1 as a novel immune prognostic biomarker for stage III colon cancer. EPSTI1 expression was positively associated with relapse-free survival and with M1-like Mϕs and myeloid DCs infiltrating TME. Akimoto et al. showed that immature desmoplastic reaction and myxoid stroma were associated with lower frequency of memory CD8^+^ T cells and M1-like Mϕs in the epithelial and stroma fractions of colorectal tumors, respectively. They described a relationship between immune and non-immune cells inside colorectal TME supporting the notion that these interactions can serve as prognostic markers.

Considering glioblastoma, Wang et al. developed a metabolic-related gene pair (MRGP) prognostic signature, that allows patient stratification into high- and low-risk groups, in terms of overall survival. The high-risk group showed a particular TME immunoscore, characterized by an increase in monocytes and fewer activated DCs, natural killer (NK) cells and gamma-delta (γδ) T cells. MRGP analysis showed that ABCA1 expression increases on tumor-associated Mϕs (TAMs) as tumor progresses, suggesting that cholesterol metabolism plays a vital role in the functional polarization of TAMs. Lovastatin treatment, both *in vitro* and *in vivo*, reduced ABCA1 expression in TAMs and promoted their polarization towards an inflammatory phenotype, which in turn controlled tumor progression. Therefore, they elected ABCA1 expressed on TAMs as a feasible prognostic biomarker for primary glioblastoma.

In the context of lung cancer, Zhao et al. investigated the clinical significance of tertiary lymphoid structure (TLS) maturity and its association with the spatial distribution of tumor-resident memory (T_RM_) T cell subsets, in advanced stage III lung adenocarcinoma (LUAD). The authors divided the patients in three groups, alongside TLS maturation state. The results showed that the proportion of CD4^+^CD103^+^ and CD8^+^CD103^+^ T_RM_ T cells, preferentially located within TLS was significantly increased by TLS gradual maturation and disease-free survival. Taking together, these authors suggested that TLS maturation state and the frequency of T_RM_ could be used as prognostic markers in stage III LUAD. In non-small cell lung cancer (NSCLC), Wang et al. showed that neutrophil extracellular traps (NETs) produced in the TME activate the NF-kB/NLRP3 pathway by downregulating MIR503HG expression to promote epithelial to mesenchymal transition (EMT) and metastasis.

Regarding tumor infiltrating lymphocytes, Chen et al. investigated the controversial prognostic value of CD8^+^ T cells infiltrating papillary thyroid cancer (PTC). Flow cytometry analysis and multiplex immunohistochemistry of tissue samples from multinodular non-toxic goiter (MNG), ─ taken as normal thyroid tissue, versus PTC tumors showed that CD8^+^ T cells in cancer patients present a dysfunctional state. Importantly, compared data from immune cells status, during the process of MNG to PTC, showed that the CD8^+^ T cell immune contexture is altered after the occurrence of malignancy, moving from an activated anti-tumor status toward an inhibitory phenotype. Savid-Frontera et al. analyzed the role of virtual memory CD8^+^ T (T_VM_) cells, ─ a T cell subset with innate-like characteristics-, for anti-tumor immune response. They showed that systemic expression of both IL-12 and IL-18 in experimental models of melanoma and pancreatic ductal adenocarcinoma leads to an early accumulation of T_VM_ cells in the TME, which contribute to control tumor growth.

Liu et al. published a review concerning the relationship between programmed cell death and the modulation of TME towards an immunosuppressive profile. Bolesina et al. reported a correlation between heavy alcohol consumption and a higher TLR9 expression on oral squamous cell carcinoma (OSCC). OSCC expressing TLR9 molecules showed a decrease in tumor infiltrating CD8^+^ T cells and a diminished overall survival. These authors suggest that the loop generated by alcohol consumption and a higher TLR9 expression might have an impact on the distribution of CD8^+^ T cells inside the tumor and patient survival. Jiang et al. showed that in patients with renal cell carcinoma the expression of methyltransferase METTL24 was lower in tumors than in adjacent normal tissue. METTL24 expression correlated with several immune parameters, many linked to NF-κB signaling and a low METTL24 expression correlated with poor prognosis.

Altogether, these articles highlight the progress in our understanding of how immunophenotypes infiltrating the TME regulate many facets of tumor development and progression, but also help underscore many unresolved and even controversial areas of tumor immunology research. We are grateful for the considerable efforts that the authors and reviewers have made to help us compile this collection for Frontiers in Immunology.

## Author contributions

ACM: Drafted the article; APL, MB and MF: provided critical inputs and corrected the manuscript. All authors contributed to the article and approved the submitted version.

